# Left atrial volumetric and functional remodeling post-pulmonary vein isolation: Insights from cardiac magnetic resonance imaging

**DOI:** 10.1016/j.jocmr.2025.101937

**Published:** 2025-08-06

**Authors:** Nikki van Pouderoijen, Luuk H.G.A. Hopman, Leontine E. Wentrup, Joris R. de Groot, Michiel J.B. Kemme, Cornelis P. Allaart, Marco J.W. Götte

**Affiliations:** Department of Cardiology, Amsterdam UMC, Amsterdam, the Netherlands

**Keywords:** Atrial fibrillation, Cardiac MRI, Pulmonary vein isolation, Left atrial strain, Left atrial reverse remodeling

## Abstract

**Background:**

Atrial fibrillation (AF) ablation may induce reverse left atrial (LA) remodeling, yet few studies have prospectively evaluated its short- and long-term effects. This study assessed LA volumetric and functional remodeling using cardiovascular magnetic resonance (CMR) imaging early and late after pulmonary vein isolation (PVI) in AF patients.

**Methods:**

This study involved 61 AF patients undergoing radiofrequency PVI. CMR scans were performed pre-PVI, within 72 h and 3 months post-PVI. LA volumes and strain were assessed using two- and four-chamber cine images. Early AF recurrence was monitored during 3 months follow-up.

**Results:**

LAVImin significantly increased early post-PVI (22.5±8.7 mL/m² to 25.8±9.9 mL/m², p<0.01). At 3 months, both LAVImin and LAVImax significantly reduced compared to early post-PVI (25.4±8.87 mL/m^2^ to 19.4±7.7 mL/m^2^, p<0.001; 48.2±12.7 mL/m^2^ to 38.7±10.6 mL/m^2^, p<0.001, respectively), as well as compared to baseline (22.5±8.7 mL/m^2^ to 20.1±8.5 mL/m^2^, p = 0.04; 45.6±11.8 mL/m^2^ to 39.3±11.2 mL/m^2^, p<0.001, respectively). Early post-PVI, LA emptying fraction (LA EF), LA reservoir, and contractile strain significantly reduced compared to baseline (from 51.6±10.8% to 47.1±8.9%, p<0.01; 18.3±4.4% to 15.4±2.9%, p<0.001; 8.3±3.1% to 5.4±1.8%, p<0.001, respectively). At 3 months, LA EF, LA reservoir, and contractile strain significantly increased as compared to early post-PVI (from 47.1±8.9% to 50.5±8.6%, p<0.01; 15.4±2.9% to 16.8±3.1%, p<0.01; 5.4±1.8% to 6.9±2.3%, p<0.001, respectively). However, LA reservoir and contractile strain remained significantly lower compared to baseline (18.3±4.4% to 16.8±3.1%, p = 0.02; 8.3±3.1% to 6.9±2.3%, p<0.01, respectively).

In patients with early AF recurrence 27.9% (17/61), LA volume reduction and partial functional recovery were not observed during 3 months post-PVI.

**Conclusion:**

LA volumes significantly reduced 3 months post-PVI. While LA function initially declined, it showed partial recovery at 3 months. However, LA reservoir and contractile strain remained reduced compared to pre-PVI. LA reverse remodeling and partial LA functional recovery only occurred in patients without early AF recurrence.

## Highlights

• LA volumes initially increased, likely due to acute ablation effects, but decreased by 3 months, indicating reverse remodeling.
• Early post-PVI, LA function declined with partial recovery by 3 months, yet remained impaired, likely due to ablation scar.
• LA reverse remodeling and partial LA functional recovery only occurred in patients without early AF recurrence.

## Graphical abstract

**Figure:**
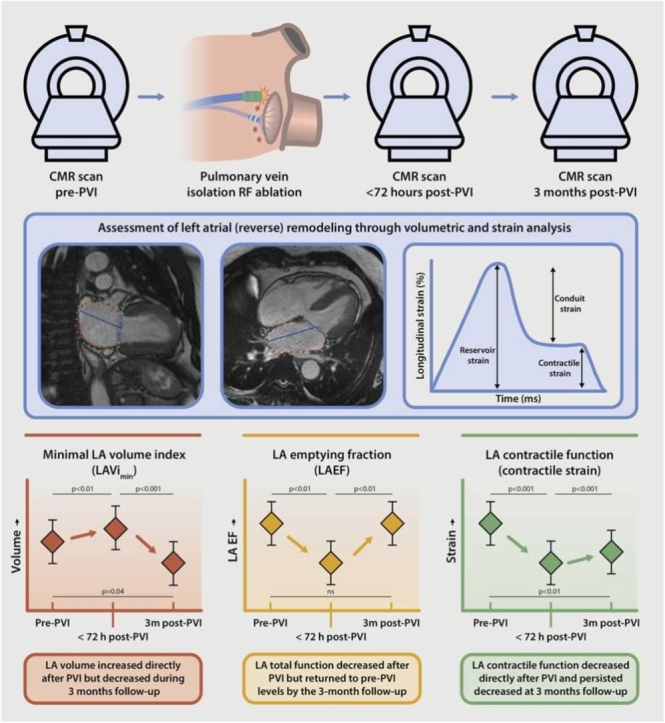

## 1. Introduction

Pulmonary vein isolation (PVI) is a well-established treatment strategy for catheter ablation in patients with atrial fibrillation (AF) [1], [2]. Radiofrequency (RF) ablation is the most frequently used ablation technique, creating acute coagulation necrosis through thermal mechanisms. This results in immediate histological changes, including atrial microvascular injury and edema during the early post-ablation phase [3], [4]. Over time, these acute lesions evolve into areas of reparative fibrosis, ultimately forming scar tissue surrounding the pulmonary veins post-ablation [5].

Modification of left atrial (LA) tissue by ablation can lead to dynamic changes in LA volumes and function. Immediately following PVI, LA function may be compromised due to the acute inflammatory response and subsequent edema formation resulting from the ablation procedure [6]. Also in a later stage, LA function may remain impaired due to the replacement of atrial myocytes with non-contractile scar tissue [7], [8]. Significant atrial scarring following PVI can lead to increased LA stiffness. Excessive stiffness has been linked to adverse remodeling of the LA and may play a crucial role in the development of various cardiovascular conditions such as heart failure [9], [10]. Successful restoration of sinus rhythm through PVI, on the other hand, has been shown to result in improved LA function and reduced LA volumes [11], [12]. This reverse remodeling is considered to result from eliminating or diminishing AF burden, allowing the LA to recover and reduce its size.

Current understanding of LA remodeling following ablation is largely derived from a limited number of echocardiographic studies with minimal insight into the remodeling processes that have been studied systematically in an early and 3-month stage after PVI [12], [13]. However, cardiovascular magnetic resonance imaging (CMR) remains the gold standard for accurate assessment of LA volumes and function. Especially, feature tracking strain analysis by CMR provides a robust method to evaluate atrial function across the three atrial phases (i.e., reservoir, conduit, and contractile), offering a detailed understanding of atrial mechanics throughout the cardiac cycle [8]. Currently, there are few studies that have prospectively assessed the short- and long-term impact of RF ablation on LA remodeling and detailed mechanics [14], [15].

Therefore, this study aims to investigate the dynamic changes in LA volumes and function using CMR during the early (<72 h) and late (3 months) stages after RF PVI ablation in AF patients.

## 2. Method

### 2.1. Study design

This is a prospective single-center cohort study that was conducted according to the principles outlined in the 1964 Declaration of Helsinki and its later amendments. Collection and management of data were approved by the local medical ethics committee (Amsterdam University Medical Center, Amsterdam, The Netherlands). Written informed consent was obtained from all patients included in this study.

### 2.2. Study population

**Fig. 1:**
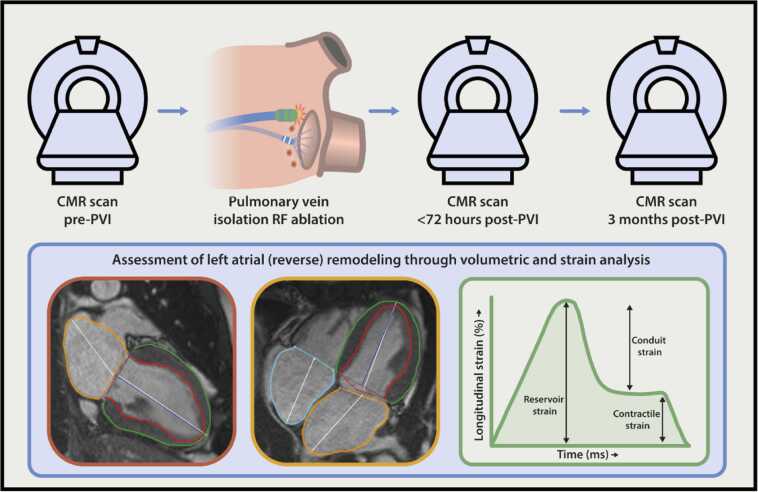
Schematic illustration of the study methodology. The present study included AF patients undergoing primo RF PVI and three sequential CMR scans: prior to PVI, and <72 h and 3 months post-PVI. LA and LV volumes and function were derived from the two-chamber and four-chamber SSFP cine images using the biplanar method. Automatic contouring was performed in the atrial end-diastolic and end-systolic phase and manual adjustments were made when deemed necessary. Longitudinal strain analysis was performed using the feature tracking module in Circle CVI^42^ (version 5.11.4; Cardiovascular Imaging, Inc, Calgary, Alberta, Canada). Endocardial and epicardial borders of the LA and LV were segmented in the end-diastolic and end-systolic phase of the long-axis cine images. LA strain was subdivided by the reservoir, conduit, and contractile strain. *AF* atrial fibrillation, *PVI* pulmonary vein isolation, *LV* left ventricle, *CMR* cardiovascular magnetic resonance imaging, *LA* left atrial, *PVI* pulmonary vein isolation, *RF* radiofrequency, *SSFP* steady-state free precession

Patients with paroxysmal or persistent AF meeting the HRS/EHRA guideline criteria and referred for primary RF PVI ablation procedure were enrolled in this study [1]. As part of routine clinical work-up, patients underwent a pre-PVI CMR scan to evaluate PV anatomy, exclude LA appendage (LAA) thrombus, and assess cardiac function. Exclusion criteria for participation in the study were the standard contraindications for CMR, AF during the pre-PVI CMR scan, a history of LA ablation, cardiac surgery, or chest radiation therapy. All included patients underwent two sequential post-PVI CMR scans, one early (within 72 h) after, and one late (at 3 months) after ablation (Fig. 1). Only patients who were in sinus rhythm during each CMR scan were included in the study’s (sub)analyses.

### 2.3. Ablation protocol

All RF ablation procedures were performed under general anesthesia. RF ablation lesions were created to isolate the PVs by a point-by-point approach using a contact force-sensing ablation catheter or single-shot RF balloon catheter (SmartTouch, QDOT, or HELIOSTAR, Biosense Webster, Diamond Bar, California). Durability of complete PV isolation was assessed by bidirectional conduction block after a waiting period of at least 20 min. No additional LA ablation lines were placed.

### 2.4. Recurrence during 3 months of follow-up

Patients were monitored for 3 months following PVI, with rhythm assessments conducted through 24-h Holter monitoring and additional electrocardiogram (ECG) or Kardia (Alivecor, Mountain View, California) recordings on indication in patients experiencing symptoms suggestive of tachyarrhythmia recurrence. Antiarrhythmic drugs were continued for 3 months following the ablation procedure. Any documented episode of AF, atrial flutter, or atrial tachycardia lasting >30 s during 90 days post-ablation was considered early AF recurrence.

### 2.5. CMR acquisition protocol

All CMR scans were performed using a 1.5T clinical MRI scanner (Sola, Siemens Healthineers, Erlangen, Germany) with a dedicated phased array cardiac receiver coil. The scan protocol included retrospective ECG‐gated steady‐state free precession cine imaging with breath‐holding in standard two-chamber and four-chamber long‐axis views for the assessment of LA and LV function and volumes. The acquisition parameters were as follows: echo time of 1.6 ms, slice thickness of 5 mm, flip angle of 60°–75°, matrix of 256 × 208 mm, and temporal resolution of more than 30 ms.

### 2.6. CMR analysis

The acquired two-chamber and four-chamber cine images were post-processed using Circle CVI^42^ (version 5.11.4; Cardiovascular Imaging, Inc., Calgary, Alberta, Canada).

#### 2.7. Volume assessment

LA and LV volumes were obtained from the two-chamber and four-chamber cine images using the biplanar method. Automatic contouring was performed in the atrial end-diastolic and end-systolic phase and manual adjustments were made when deemed necessary (Fig. 1). LA emptying fraction (LA EF) was calculated using the LA maximal volume (LAVmax) and LA minimal (LAVmin) volume. LAVmax and LAVmin were indexed to body surface area (BSA), LAVImax and LAVImin, respectively. LV ejection fraction (LVEF) was calculated using the LV end-diastolic (EDV) and end-systolic volume (ESV).

#### 2.8. Longitudinal strain

Longitudinal strain analysis was performed using the feature tracking module in Circle CVI^42^. Endocardial and epicardial borders of the LA and LV were automatically segmented in the end-diastolic phase of the two- and four-chamber view long-axis cine images. Proper atrial and ventricular wall tracing was verified using a Mesh overlay, with manual adjustments applied if necessary. LA strain was subdivided into the reservoir, conduit, and contractile strain (Fig. 1).

### 2.9. Statistical analysis

Continuous variables were expressed as mean±standard deviation for normally distributed data and median and interquartile range (IQR) for skewed data. Categorical variables were expressed as frequency and percentage. Differences in LA parameters across time points were analyzed using repeated measures analysis of variance (ANOVA) for normally distributed data or the Friedman test for non-parametric data, with Bonferroni-corrected pairwise comparisons applied in both cases. Comparisons between two groups were made with the independent samples T-test for normally distributed data or Mann-Whitney U test for non-parametric data. Statistical significance was defined as a p-value <0.05. For multiple comparisons between groups, the Bonferroni correction was applied, adjusting the significance threshold to p-values <0.017. Statistical analysis was performed using SPSS Statistics, version 28 (IBM Corp, Armonk, New York).

## 3. Results

### 3.1. Study population

This study included 61 patients who underwent a primary PVI procedure. Baseline characteristics are presented in Table 1. The mean age was 61±8 years, 73.8% (45/61) were male, and 70.5% (43/61) had paroxysmal AF prior ablation. The pre-PVI MRI was conducted 67 [41−128] days before PVI, while the early and late post-PVI MRI were performed 24 [24–72] hours, and 93 [91−98] days after PVI, respectively.

**Table 1:** Baseline patient characteristics

N = 61	
Age, y	60.7±7.8
Male, n (%)	45 (73.8%)
BMI, kg/m^2^	26.7±4.2
eGFR (mL/min/1.73m^2^)	79.5±12.8
CHA_2_DS_2_-VASc ≥ 2	19 (31.1%)

*AF type, n (%)*	
Paroxysmal AF	43 (70.5%)
Persistent AF	18 (29.5%)
AF duration (mo)	48[21–88]

*Medical history, n (%)*	
Hypertension	10 (16.4%)
Diabetes Mellitus	3 (4.9%)
Coronary artery disease	4 (6.6%)
CVA/TIA	6 (9.8%)
Sleep apnea	7 (11.9%)
*Medication, n (%)*	
Amiodarone	8 (13.1%)
Flecainide	26 (42.6%)
Sotalol	14 (23%)
Metoprolol	15 (24.6%)
Verapamil	12 (19.7%)
None	4 (6.6%)
Anticoagulation - Rivaroxaban - Edoxaban - Dabigatran - Apixaban - Vitamin K antagonist	61 (100%) 24 (39.3%) 16 (26.2%) 7 (11.5%) 13 (21.3%) 1 (1.6%)

Data are expressed as mean±SD, median (interquartile range), or number (percentage). *AF* atrial fibrillation, *BMI* body mass index, *CVA* cerebral vascular accident, *eGFR* estimated glomerular filtration rate, *SD* standard deviation, *TIA* transient ischemic attack

### 3.2. Early and late impact of RF ablation

#### 3.2.1. Early impact post-PVI

**Fig. 2:**
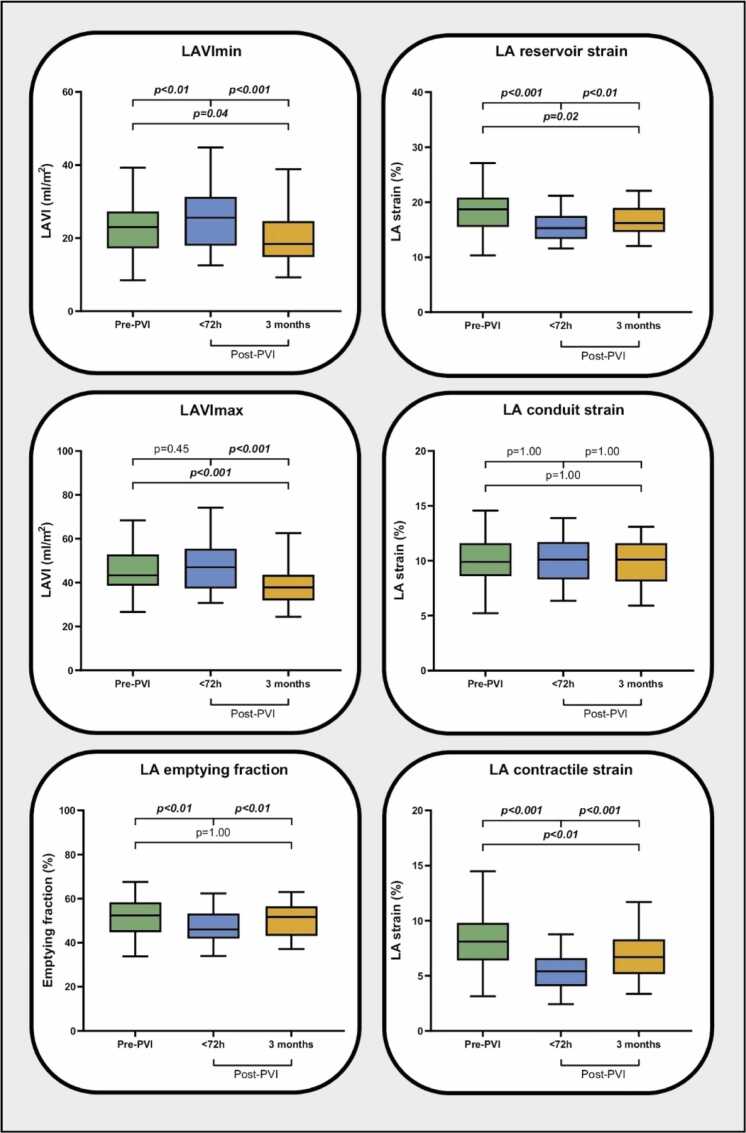
LA volumes and function before PVI, and <72 h and 3 months after PVI. Differences in LAVImin, LAVImax, LA EF, LA reservoir strain, LA conduit strain, and LA contractile strain before PVI, and <72 h and 3 months after PVI. *LAVI* left atrial volume index, *PVI* pulmonary vein isolation, *LA* left atrial, *EF* emptying fraction

Early post-PVI, LAVImin significantly increased from 22.5±8.7 mL/m² to 25.8±9.9 mL/m² (p<0.01), while LAVImax remained unchanged compared to baseline (45.6±11.8 mL/m² to 47.8±12.6 mL/m^2^, p = 0.45 (Fig. 2, Table 2). During this stage, LA EF, LA reservoir strain, and LA contractile strain were significantly decreased (from 51.6±10.8% to 47.1±8.9%, p<0.01; 18.3±4.4% to 15.4±2.9%, p<0.001; 8.3±3.1% to 5.4±1.8%, p<0.001, respectively), whereas LA conduit strain showed no significant difference (10.0±2.5% to 10.0±2.2%, p = 1.00).

**Table 2:** LA and LV volumes and function before and after PVI

	Pre-PVI N = 61	<72 h post-PVI N = 61	3 months post-PVI N = 61	Pre to <72 h post-PVI	Pre to 3 months post-PVI	<72 h to 3 months post-PVI
*LA parameters*						
LA reservoir strain, %	18.3±4.4	15.4±2.9	16.8±3.1	<0.001	0.02	<0.01
LA conduit strain, %	10.0±2.5	10.0±2.2	9.9±2.2	1.00	1.00	1.00
LA contractile strain, %	8.3±3.1	5.4±1.8	6.9±2.3	<0.001	<0.01	<0.001
LAVImax, mL/m^2^	45.6±11.8	47.8±12.6	39.3±11.2	0.45	<0.001	<0.001
LAVImin, mL/m^2^	22.5±8.7	25.8±9.9	20.1±8.5	<0.01	0.04	<0.001
LA EF, %	51.6±10.8	47.1±8.9	50.5±8.6	<0.01	1.00	<0.01
*LV parameters*						
LV ESV, mL	78.9±20.1	81.2±25.7	79.2±22.9	0.72	1.00	0.88
LV EDV, mL	185.4±39.3	185.5±40.8	185.1±45.7	1.00	1.00	1.00
LVEF, %	57.5±5.9	56.8±6.1	57.3±5.9	0.92	1.00	1.00
LV strain, %	16.4±2.4	16.4±2.2	15.8±2.3	1.00	0.54	0.28

Data are expressed as mean±SD. p-values have been adjusted by the Bonferroni correction for multiple tests. *EDV* end-diastolic volume, *ESV* end-systolic volume, *LV* left ventricle, *LAVI* left atrial volume index, *EF* ejection/emptying fraction, *PVI* pulmonary vein isolation

#### 3.2.2. Late impact post-PVI

At 3 months post-PVI, both LAVImin and LAVImax were significantly reduced compared to early post-PVI (Fig. 2, Table 2) (25.8±9.9 mL/m^2^ to 20.1±8.5 mL/m^2^, p<0.001; 47.8±12.6 mL/m^2^ to 39.3±11.2 mL/m^2^, p<0.001), as well as compared to baseline (22.5±8.7 mL/m^2^ to 20.1±8.5 mL/m^2^, p = 0.04; 45.6±11.8 mL/m^2^ to 39.3±11.2 mL/m^2^, p<0.001, respectively). LA EF and both LA reservoir and LA contractile strain demonstrated significant improvements compared to the early post-PVI stage (from 47.1±8.9% to 50.5±8.6%, p<0.01; 15.4±2.9% to 16.8±3.1%, p<0.01; 5.4±1.8% to 6.9±2.3%, p<0.001, respectively). However, LA reservoir and LA contractile strain remained significantly lower than pre-PVI (18.3±4.4% to 16.8±3.1%, p = 0.02; 8.3±3.1% to 6.9±2.3%, p<0.01, respectively). LA EF did not significantly differ between pre-PVI and 3 months post-PVI (51.6±10.8% to 50.5±8.6%, p = 1.00) (Fig. 2, Table 2). LA conduit strain remained consistent across all PVI stages (from 10.0±2.5% to 10.0±2.2% to 9.9±2.2%, all comparisons p = 1.00). The ratio of conduit to contractile strain significantly increased early after PVI (1.5±1.0 to 2.1±1.0, p<0.001), and subsequently decreased at 3 months (2.1±1.0 to 1.6±0.7, p<0.001) (Table S5).

#### 3.2.3. Left ventricle

There were no significant differences observed in LV ESV, LV EDV, LVEF, and LV strain between pre-PVI and follow-up after PVI (Table 2).

### 3.3. Early AF recurrence

**Fig. 3:**
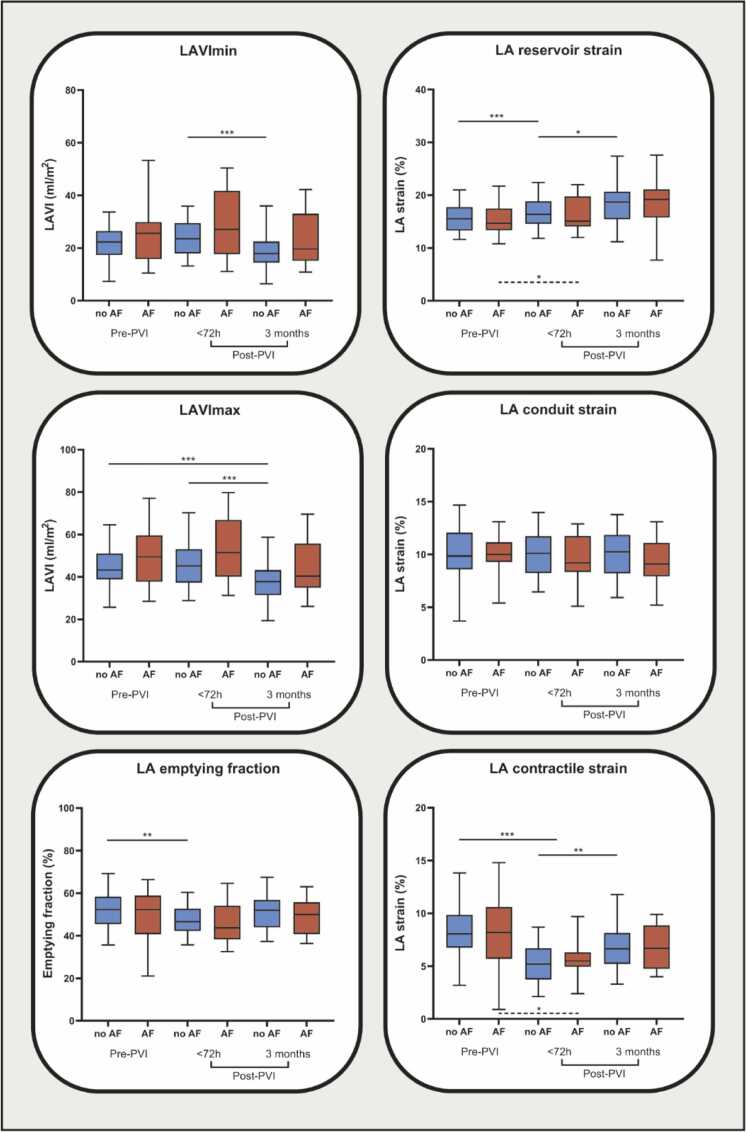
LA volumes and function before, <72 h after, and 3 months after PVI between patients with (red) and without (blue) early AF recurrence. Differences in LAVImin, LAVImax, LA EF, LA reservoir strain, LA conduit strain, and LA contractile strain of patients with (red, n = 17) and without (blue, n = 44) early AF recurrence before PVI, and <72 h and 3 months after PVI. *p<0.05; **p<0.01; ***p<0.001. *LA* left atrial, *PVI* pulmonary vein isolation, *AF* atrial fibrillation, *LAVI* left atrial volume index, *EF* ejection/emptying fraction

Arrhythmia recurrence within 90 days post-PVI was observed in 17 of the 61 patients (27.9%). Differences in baseline parameters between patients with (n = 17) and without (n = 44) early AF recurrence is presented in Supplemental Table S1. Differences in LA remodeling parameters are presented in Fig. 3 and Supplemental Tables S2, S3, and S4. There were no significant differences in LA volumes and function at baseline or during follow-up between patients with and without early AF recurrence (Fig. 3, Table S2).

LAVImin and LAVImax did not significantly decrease in the early AF recurrence group following PVI, whereas patients without early AF recurrence showed a significant reduction in both at 3 months compared to early post-PVI (Fig. 3, Tables S3 and S4). While both patient groups exhibited significantly reduced LA reservoir and LA contractile strain early after PVI compared to baseline, only patients without early AF recurrence demonstrated subsequent improvement in LA function—reflected by increases in LA reservoir strain, LA contractile strain, and a trend toward higher LA EF at 3 months post-PVI—changes not observed in patients with early AF recurrence (Fig. 3, Table S2).

## 4. Discussion

The present study evaluated LA (reverse) remodeling parameters during the early (<72 h) and late (3 months) stages after RF PVI in AF patients, as assessed by CMR. The main findings of the present study are (1) RF PVI initially led to an increase in LA volume, which was followed by a subsequent decrease at 3 months, indicating reverse remodeling occurring later after ablation; (2) LA EF decreased immediately after PVI but returned to pre-PVI levels by the 3-month follow-up; (3) LA reservoir and contractile strain were impaired early after ablation, but partially restored by 3 months, although both remained lower compared to pre-PVI; (4) LA reverse remodeling and partial LA functional recovery only occurred in patients without early AF recurrence.

### 4.1. Atrial function and remodeling after PVI

There is a well-established relationship between LA enlargement and AF, both conditions enhancing each other, and adversely affecting LA function [16], [17]. Successful restoration of sinus rhythm through catheter ablation can result in reverse remodeling of the LA, leading to a reduction in LA volume and improvement of function [11], [12]. Yet, little is known about the progression of this remodeling process after ablation and while AF may be resolved, the effects of catheter ablation per se on acute and long-term damage to the LA.

During RF ablation, lesions are created by modifying the atrial myocardium using thermal energy. This myocardial damage induced by RF may directly impact the LA volumetric and functional condition. However, data regarding acute changes in LA volumes and function after PVI are controversial and mainly rely on echocardiographic assessments [12], [13]. To our knowledge, only two studies used CMR to assess LA volumes and function in AF patients acutely (within one day) after RF PVI [14], [15]. The findings of the present study align with these previous studies by demonstrating a reduction in LA function in the acute phase after PVI [14], [15]. Moreover, the current study observed an increase in LAVImin shortly after ablation, similar to the study by Csécs and colleagues [14]. The increase in LA volume, combined with the immediate decline in LA function, as evidenced by reductions in LA EF, LA reservoir, and LA contractile strain, may result from the acute ablation effects such as myocardial injury and LA wall edema [6], [18], [19]. AF ablation triggers a cascade of inflammatory and injury responses within days, potentially affecting not only the ablated myocardium but the entire LA, which thus contributes to immediate functional deterioration [20]. The primary cause of this direct LA functional decline remains unclear, but both thermal ablation injury, which could also impact the contractile function of non-ablated myocytes, and the subsequent inflammatory response of LA tissue may play a role.

The current study is also in line with the majority of research on late LA remodeling after PVI, demonstrating a reduction in LA volume, which is evidence of reverse remodeling [14], [15], [21], [22], [23]. Reduced LA volumes late after ablation may be attributed to the restoration of sinus rhythm, but research also indicates that the shrinkage of ablation-induced scarring may contribute to a decrease in LA size over time [15], [24], [25]. At 3 months after PVI, LA function partially restored, likely due to the recovery of LA tissue and a reduced AF burden. This recovery process may involve the resolution of tissue edema and local inflammation, typically occurring within one month following the procedure [19], [20], [26]. Interestingly, LA function does not return to or improve beyond its pre-PVI state within 3 months after ablation, as indicated by the persistently reduced LA reservoir and contractile strain. This observation may be attributed to the replacement of atrial myocytes with ablation-induced scar tissue, which may impair LA contractile function [7], [15], [27]. Previous studies supported this hypothesis by demonstrating a correlation between strain abnormalities and the extent of atrial fibrosis [8], [28], [29]. Notably, LA conduit strain remained preserved after ablation, indicating that passive filling function may be less affected by ablation. Given that conduit strain primarily reflects passive LV-LA interaction and no significant changes were observed in LV function, LA conduit strain appears to remain stable. In contrast, reductions in LA reservoir and contractile strain likely reflect impaired active contractile function due to ablation-induced scar formation, consistent with our observed changes in the passive-to-active strain ratio post-PVI, and supporting prior findings that active LA function may be the primary dynamic component affected by ablation, driving subsequent declines in LA reservoir function [14].

While traditional RF ablation results in myocardial scar formation and potentially leads to LA functional deterioration, the emerging ablation strategy pulsed field ablation (PFA) may offer advantages due to its unique non-thermal ablation mechanism and subsequent atrial healing process [27], [31], [32], [22]. A CMR study by Nakatani et al. demonstrated that PFA, compared to thermal ablation, results in a reparative process without fibrosis formation, which may prevent atrial stiffening, and preserve LA function [22]. As PFA emerges as a promising candidate for the future gold standard in PVI, further research is warranted to elucidate its mechanisms of LA ablation injury and recovery of LA tissue, including structural and functional reverse remodeling.

### 4.2. Reverse LA remodeling in early AF recurrence

Maintaining sinus rhythm after AF ablation is known to promote reverse LA remodeling, but the impact of early AF recurrence on this process remains unclear [30]. One could hypothesize that early recurrence of AF may influence the extent of reverse atrial remodeling after PVI. Therefore, in this study, we specifically examined the differences between groups with and without recurrence of AF in the first 90 days after PVI to assess how this factor might affect atrial remodeling outcomes.

Overall, no significant differences were observed in volumetric or functional parameters between patients with and without early AF recurrence at each time stage. When evaluating changes within each group over time, it was observed that only patients without early AF recurrence showed a reduction in LA volumes at the 3-month follow-up compared to the early post-PVI stage. In both patient groups, LA function was reduced shortly after PVI. However, after the initial decline, patients without early AF recurrence exhibited improvements in LA reservoir and contractile strain, whereas this recovery was not observed in patients with early AF recurrence. These findings suggest that the extent of LA volumetric and functional recovery may depend on recurrence of AF during 3-month follow-up [31]. Moreover, it has been shown that the timing of occurrence and burden of (early) AF recurrence may play a critical role in determining the extent of LA reverse remodeling after ablation [31]. On the other hand, atrial remodeling is a complex process driven by the response of cardiac myocytes to not only electrical, but also varying mechanical and metabolic stressors [32], [33]. Therefore, it can also be proposed that in addition to impaired reverse remodeling after ablation, also other factors such as inflammation, procedural characteristics, and autonomic triggers may contribute to early AF recurrence, possibly resulting in the persistence of adverse remodeling [1]. Future research, including longer follow-up duration and a larger sample size, is required to provide further insight into the relationship between early and late AF recurrence, including AF burden, and (impaired) LA reverse remodeling.

## 5. Limitations

Several limitations need to be considered when interpreting the results of the present study. First, the sample size was relatively small for the subgroup-analysis, which may have limited the ability to detect substantial differences between the early-recurrence and non-recurrence groups. Moreover, while rhythm monitoring was performed using a Kardia device for symptom-triggered ECG recordings, alongside 24-hour Holter monitoring at 3 months, asymptomatic AF episodes could have been missed, potentially influencing the results of the sub-analysis. Second, although volumetric analysis using a short-axis stack encompassing both atria and ventricles would offer greater accuracy than the biplane method, its integration into the MRI protocol was precluded by time limitations. Third, the 2-month interval between baseline CMR and PVI, could raise the possibility that factors like high AF burden or comorbidities contributed to adverse remodeling during this period, potentially impacting LA remodeling assessment early post-PVI. However, given the low prevalence of comorbidities in our cohort, we consider substantial progression during this 2-month period unlikely. Fourth, AF burden before and after ablation was not evaluated, which could have provided further understanding of the influence and timing of early AF recurrence on reverse remodeling. Fifth, the proportion of women in our study was below one-third of the total cohort, which may impact the generalizability of our findings to female AF patients. Last, the follow-up duration was limited to 3 months, which did not allow for the assessment of late AF recurrence. A longer follow-up period could provide valuable insights into the long-term effects of ablation, including late AF recurrence and LA reverse remodeling.

## 6. Conclusion

This study demonstrated that RF PVI initially results in an increase in LA volume, followed by a subsequent decrease beyond pre-PVI values, indicating reverse LA remodeling in a later stage after ablation. LA function temporarily reduced early post-PVI, but partially recovered by 3 months. Full recovery of LA function, however, did not occur as evidenced by the persistently reduced LA reservoir and contractile strain at 3 months follow-up as compared to baseline, possibly due to the ablation-induced formation of atrial scarring. LA reverse remodeling and partial LA functional recovery only occurred in patients without early AF recurrence.

## Funding

This study was partially supported by an institutional research grant from Johnson & Johnson MedTech Electrophysiology (Biosense Webster IIS-566).

## Author contributions

**Nikki van Pouderoijen:** Writing – original draft, visualization, project administration, methodology, investigation, formal analysis, data curation, conceptualization. **Luuk H.G.A. Hopman:** Writing – review & editing, conceptualization. **Leontine E. Wentrup:** Writing – review & editing, project administration. **Joris R. de Groot:** Writing – review & editing. **Michiel J.B. Kemme:** Writing – review & editing. **Cornelis P. Allaart:** Writing – review & editing, supervision, conceptualization. **Gotte Marco J.W. Götte:** Writing – review & editing, supervision, conceptualization.

## Declaration of competing interests

The authors declare the following financial interests/personal relationships which may be considered as potential competing interests: C.P. Allaart reports that financial support was provided by Johnson & Johnson MedTech Electrophysiology. The other authors declare that they have no known competing financial interests or personal relationships that could have appeared to influence the work reported in this paper.

## Data Availability

The data underlying this article will be shared on reasonable request to the corresponding author.
