# Supplementary material for: Left atrial volumetric and functional remodeling post-pulmonary vein isolation: Insights from cardiac magnetic resonance imaging
Source: J Cardiovasc Magn Reson. 2025 Aug 6;27(2):101937. doi: 10.1016/j.jocmr.2025.101937 (PMC12673017; doi:10.1016/j.jocmr.2025.101937)
Supplement: Supplementary file 1 — Supplementary material [file mmc1.docx]

**SUPPLEMENTS**

**Left Atrial Volumetric and Functional Remodeling Post-Pulmonary Vein Isolation:**

**Insights from Cardiac Magnetic Resonance Imaging**

**Table S1. Baseline patient characteristics between patients with and without early AF recurrence**

| **Baseline patient characteristics** | **All**  **N=61** | **No early AF recurrence**  **N=44**  **72.1%** | **Early AF recurrence**  **N=17**  **27.9%** | **P-value**  **N/Y** |  |
| --- | --- | --- | --- | --- | --- |
|  |  |  |  |  |  |
| **Age, years** | 60.7 ± 7.8 | 60.4 ± 8.5 | 61.5 ± 5.6 | 0.61 |  |
| **Male, n (%)** | 45 (73.8%) | 33 (75%) | 12 (70.6%) | 0.73 |  |
| **BMI, kg/m^2^** | 26.7 ± 4.2 | 26.6 ± 4.3 | 26.8 ± 4.3 | 0.91 |  |
| **eGFR (ml/min/1.73m^2^)** | 79.5 ± 12.8 | 79.2 ± 13.3 | 80.4 ± 11.7 | 0.74 |  |
| **CHA_2_DS_2_-VASc ≥ 2** | 19 (31.1%) | 13 (29.5%) | 6 (35.3%) | 0.66 |  |
|  | | | | |  |
| ***AF type, n (%)*** | | | | |  |
| **Paroxysmal AF** | 43 (70.5%) | 30 (68.2%) | 13 (76.5%) | 0.53 |  |
| **Persistent AF** | 18 (29.5%) | 14 (31.8%) | 4 (23.5%) | 0.53 |  |
| **AF duration (months)** | 48 [21 – 88] | 48 [21 – 87] | 53 [16 – 96] | 0.97 |  |
|  | | | | |  |
| ***Medical history, n (%)*** | | | | |  |
| **Hypertension** | 10 (16.4%) | 7 (15.9%) | 3 (17.6%) | 0.87 |  |
| **Diabetes Mellitus** | 3 (4.9%) | 2 (4.5%) | 1 (5.9%) | 0.26 |  |
| **Coronary artery disease** | 4 (6.6%) | 2 (4.5%) | 2 (11.8%) | 0.50 |  |
| **CVA/TIA** | 6 (9.8%) | 3 (6.8%) | 3 (17.6%) | 0.20 |  |
| **Sleep apnea** | 7 (11.9%) | 4 (9.1%) | 3 (17.6%) | 0.38 |  |
| ***Medication, n (%)*** | | | | |  |
| **Amiodarone** | 8 (13.1%) | 6 (13.6%) | 2 (11.8%) | 0.85 |  |
| **Flecainide** | 26 (42.6%) | 22 (50%) | 4 (23.5%) | *0.06* |  |
| **Sotalol** | 14 (23%) | 9 (20.5%) | 5 (29.4%) | 0.46 |  |
| **Metoprolol** | 15 (24.6%) | 12 (27.3%) | 3 (17.6%) | 0.43 |  |
| **Verapamil** | 12 (19.7%) | 9 (20.5%) | 3 (17.6%) | 0.81 |  |
| **None** | 4 (6.6%) | 2 (4.5%) | 2 (11.8%) | 0.31 |  |
| **Anticoagulation**   - **Rivaroxaban** - **Edoxaban** - **Dabigatran** - **Apixaban** - **VKA** | 61 (100%)  24 (39.3%)  16 (26.2%)  7 (11.5%)  13 (21.3%)  1 (1.6%) | 44 (100%)  13 (29.5%)  13 (29.5%)  6 (13.6%)  1 (5.9%)  0 (0%) | 17 (100%)  11 (64.7%)  3 (17.6%)  1 (5.9%)  12 (27.3%)  1 (5.9%) | 0.01  0.34  0.39  *0.07*  0.11 |  |
| *Data are expressed as mean ± SD, median (interquartile range), or number (percentage). Abbreviations: AF, atrial fibrillation; BMI, body mass index; CVA, cerebral vascular accident; eGFR, estimated glomerular filtration rate; SD, standard deviation; TIA, transient ischemic attack; VKA, vitamin K antagonist.* | | | | | |

**Table S2. Differences in LA volumes and function between patients with and without early AF recurrence before PVI, and <72 hours after, and 3 months after PVI**

|  | *Pre-PVI*  *No AF*  *N=44* | *Pre-PVI*  *AF*  *N=17* | *<72h*  *No AF*  *N=44* | *<72h*  *AF*  *N=17* | *3m*  *no AF*  *N=44* | *3m*  *AF*  *N=17* | *Pre-PVI*  *No AF vs. AF* | *<72h*  *No AF vs. AF* | *3m*  *No AF vs. AF* |
| --- | --- | --- | --- | --- | --- | --- | --- | --- | --- |
| LA analysis (n=61) | | | | | | | | | |
| LA reservoir strain, % | 18.7 [15.5 – 20.7] | 19.2 [15.8 – 21.1] | 15.5 [13.3 – 17.7] | 14.7 [13.4 – 17.5] | 16.4 [14.6 – 18.9] | 15.1 [14.1 – 19.8] | 0.93 | 0.78 | 0.31 |
| LA conduit strain, % | 9.9 [8.6 – 12.1] | 10.0 [9.3 – 11.2] | 10.1 [8.3 – 11.7] | 9.2 [8.4 – 11.8] | 10.3 [8.2 – 11.9] | 9.1 [8.0 – 11.1] | 0.90 | 0.72 | 0.47 |
| LA contractile strain, % | 8.1 [6.8 – 9.9] | 8.2 [5.7 – 10.6] | 5.2 [3.8 – 6.7] | 5.5 [5.0 – 6.3] | 6.7 [5.2 – 8.2] | 6.7 [4.8 – 8.9] | 0.99 | 0.71 | 0.98 |
| LAVImax, mL/m^2^ | 43.2 [38.9 – 51.0] | 49.4 [37.8 – 59.6] | 45.2 [37.3 – 53.0] | 51.4 [40.2 – 66.8] | 37.8 [31.5 – 43.2] | 40.3 [35.0 – 55.7] | 0.35 | 0.15 | 0.21 |
| LAVImin, mL/m^2^ | 22.3 [17.3 – 26.4] | 25.6 [15.8 – 29.8] | 23.5 [18.0 – 29.4] | 27.0 [17.7 – 41.7] | 17.9 [14.5 – 22.5] | 19.6 [15.2 – 33.0] | 0.30 | 0.18 | 0.26 |
| LA EF, % | 52.3 [45.5 – 58.4] | 52.3 [40.7 – 58.8] | 46.6 [42.3 – 52.7] | 43.7 [38.4 – 54.1] | 52.0 [44.0 – 56.8] | 50.0 [40.8 – 55.8] | 0.77 | 0.48 | 0.27 |

*Data are expressed as median (interquartile range). For multiple comparisons between groups, the Bonferroni correction was applied, adjusting the significance threshold to p-values <0.017. Abbreviations reported in previous tables/figures*

**Table S3. LA volumes and function before PVI, and <72 hours after, and 3 months after PVI in patients without early AF recurrence**

|  | Pre-PVI | <72h  post-PVI | 3 months post-PVI | Pre to <72h post-PVI | Pre to 3 months post-PVI | <72h to 3 months post-PVI |
| --- | --- | --- | --- | --- | --- | --- |
| LA analysis (n=44)  No early recurrence | | | | | | |
| LA reservoir strain, % | 18.7 [15.5 – 20.7] | 15.5 [13.3 – 17.7] | 16.4 [14.6 – 18.9] | <0.01 | 0.09 | 0.01 |
| LA conduit strain, % | 9.9 [8.6 – 12.1] | 10.1 [8.3 – 11.7] | 10.3 [8.2 – 11.9] | 1.00 | 1.00 | 0.93 |
| LA contractile strain, % | 8.1 [6.8 – 9.9] | 5.2 [3.8 – 6.7] | 6.7 [5.2 – 8.2] | <0.01 | *0.06* | 0.001 |
| LAVImax, mL/m^2^ | 43.2 [38.9 – 51.0] | 45.2 [37.3 – 53.0] | 37.8 [31.5 – 43.2] | 1.00 | <0.01 | <0.01 |
| LAVImin, mL/m^2^ | 22.3 [17.3 – 26.4] | 23.5 [18.0 – 29.4] | 17.9 [14.5 – 22.5] | 0.13 | *0.06* | <0.01 |
| LA EF, % | 52.3 [45.5 – 58.4] | 46.6 [42.3 – 52.7] | 52.0 [44.0 – 56.8] | <0.01 | 0.86 | *0.06* |
| *Data are expressed as median (interquartile range). P-values have been adjusted by the Bonferroni correction for multiple tests. Abbreviations reported in previous tables/figures.* | | | | | | |

**Table S4. LA volumes and function before PVI, and <72 hours after, and 3 months after PVI in patients with early AF recurrence**

|  | Pre-PVI | <72h  post-PVI | 3 months post-PVI | Pre to <72h post-PVI | Pre to 3 months post-PVI | <72h to 3 months post-PVI |
| --- | --- | --- | --- | --- | --- | --- |
| LA analysis (n=17)  Early AF recurrence | | | | | | |
| LA reservoir strain, % | 19.2 [15.8 – 21.1] | 14.7 [13.4 – 17.5] | 15.1 [14.1 – 19.8] | 0.02 | 1.00 | 0.18 |
| LA conduit strain, % | 10.0 [9.3 – 11.2] | 9.2 [8.4 – 11.8] | 9.1 [8.0 – 11.1] | 1.00 | 1.00 | 1.00 |
| LA contractile strain, % | 8.2 [5.7 – 10.6] | 5.5 [5.0 – 6.3] | 6.7 [4.8 – 8.9] | 0.04 | 0.51 | 0.80 |
| LAVImax, mL/m^2^ | 49.4 [37.8 – 59.6] | 51.4 [40.2 – 66.8] | 40.3 [35.0 – 55.7] | 1.00 | 0.18 | *0.08* |
| LAVImin, mL/m^2^ | 25.6 [15.8 – 29.8] | 27.0 [17.7 – 41.7] | 19.6 [15.2 – 33.0] | 1.00 | 0.51 | 0.26 |
| LA EF, % | 52.3 [40.7 – 58.8] | 43.7 [38.4 – 54.1] | 50.0 [40.8 – 55.8] | 0.26 | 1.00 | 0.51 |
| *Data are expressed as median (interquartile range). P-values have been adjusted by the Bonferroni correction for multiple tests. Abbreviations reported in previous tables/figures.* | | | | | | |

**Table S5. LA strain ratios before PVI, and <72 hours after, and 3 months after PVI**

|  | Pre-PVI  N=61 | <72h  post-PVI  N=61 | 3 months  post-PVI  N=61 | Pre to <72h post-PVI | Pre to 3 months post-PVI | <72h to 3 months post-PVI |
| --- | --- | --- | --- | --- | --- | --- |
| LA strain ratios | | | | | | |
| Reservoir/Conduit | 1.9 ± 0.5 | 1.6 ± 0.2 | 1.8 ± 0.4 | <0.001 | 0.11 | <0.001 |
| Reservoir/Contractile | 2.5 ± 1.0 | 3.1 ± 1.0 | 2.6 ± 0.7 | <0.001 | 0.81 | <0.001 |
| Conduit/Contractile | 1.5 ± 1.0 | 2.1 ± 1.0 | 1.6 ± 0.7 | <0.001 | 0.81 | <0.001 |
| *Data are expressed as mean ± SD. P-values have been adjusted by the Bonferroni correction for multiple tests. Abbreviations reported in previous tables/figures.* | | | | | | |
